# Short-term intake of a Japanese-style healthy lunch menu contributes to prevention and/or improvement in metabolic syndrome among middle-aged men: a non-randomized controlled trial

**DOI:** 10.1186/1476-511X-13-57

**Published:** 2014-03-27

**Authors:** Hiroko Inoue, Ryosuke Sasaki, Izumi Aiso, Toshiko Kuwano

**Affiliations:** 1 Department of Food and Nutritional Sciences and Environmental Health Sciences, Graduate School of Integrated Pharmaceutical and Nutritional Sciences, University of Shizuoka, 52-1 Yada, Suruga-ku, Shizuoka 422-8526, Japan

**Keywords:** Workplace cafeteria, Metabolic syndrome, Japanese-style healthy lunch menu, Blood pressure, Serum lipids

## Abstract

**Background:**

Metabolic syndrome is now widely appreciated as a cluster of metabolic abnormalities such as visceral obesity, hypertension, hyperglycemia and dyslipidemia. To date, incidence of metabolic syndrome is continuously increasing worldwide.

In addition, low vegetable consumption has recently become a serious issue in Japan. Furthermore, Japan is facing a shortfall in places offering food that can help prevent metabolic syndrome in the first place. Our study is designed to influence these developments. We conducted a non-randomized controlled trial by offering a Japanese-style healthy lunch menu to middle-aged men in a workplace cafeteria. This menu was designed to prevent and reduce metabolic syndrome.

**Methods:**

This intervention study took the form of a non-randomized controlled trial. Participants chose the control or intervention group. The control group consumed their habitual lunches without restriction and only nutrient contents were assessed. The intervention group received a Japanese-style healthy lunch at a workplace cafeteria for 3 months. The participants worked in offices at a city hall and mostly had low levels of physical activity. Data of 35 males (control group: 7 males, intervention group: 28 males, mean age: 47.2 ± 7.9 years) were collected and analyzed.

**Results:**

We obtained an effective outcome by demonstrating that ongoing intake of a Japanese-style healthy lunch decreased blood pressure and serum lipids and increased plasma ghrelin levels. The results grew more pronounced as intake of Japanese-style healthy lunches increased in frequency.

**Conclusions:**

This study presents new empirical data as a result of an original intervention program undertaken in Japan. A Japanese-style healthy lunch menu containing many vegetables consumed can help prevent and/or improve metabolic syndrome.

## Background

Washoku (Japanese-style diet) was recognized as a cultural treasure by UNESCO in 2013. A Japanese-style diet features a grain dish (syusyoku in Japanese), side vegetable dishes (fukusai in Japanese), and a main meat or fish dish (syusai in Japanese), which offer balanced nutrition [1]. The Japanese-style diet contains little animal fat and is a healthy diet.

On the other hand, metabolic syndrome, which is closely associated with abdominal obesity, type 2 diabetes, and cardiovascular disease (CVD), has become a global epidemic in developed nations, including Japan [2,3]. Metabolic syndrome is a cluster of metabolic abnormalities characterized by concurrent hyperglycemia, hypertension, high triacylglycerol (TG) levels, low high-density lipoprotein cholesterol (HDL-Chol) levels, and inflammation [4,5]. These major components are often associated with decreased insulin sensitivity, proinflammatory, pro-oxidant, and prothrombotic states, and low levels of cardiorespiratory fitness [5]. The National Health Promotion Campaigns for the 21^st^ Century (Healthy Japan 21), supported by the Japanese Ministry of Health, Labour and Welfare (2000) recommends that adults ingest ≥ 350 g of vegetables per day [6]. However, all age groups in Japan are falling short of this goal [7]. Some intervention studies focusing on vegetable intake to prevent metabolic syndrome and improve abdominal obesity have been undertaken, but few have taken place in Japan [8]. The Mediterranean diet has been studied in detail [9-11], and some intervention studies have examined the effects of consuming low-carbohydrate and low-fat diets [12]. Epidemiological surveys have applied principal component analysis to investigations into metabolic syndrome, CVD, and associations between food consumption patterns and metabolic syndrome [13,14].

We conducted the first intervention study by providing a Japanese-style healthy lunch menu to middle-aged men at a workplace cafeteria in order to prevent and/or improve metabolic syndrome.

## Methods

### Participants

The study participants work in an office in the S city hall and none partake in daily exercise. They only engage in desk work and commute by train or a bus. Given the office-bound nature of their jobs, even mild activity such as walking in the course of work is minimal.

### Sample selection

After explaining the study aim and procedure to the entire office, we enrolled sufficient participants to begin. A recruitment period of the study participants was one month.

**Figure 1:**
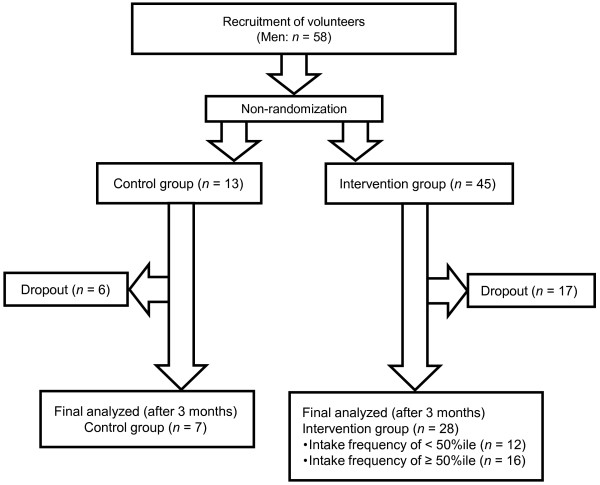
**Flow diagram of study participants.** Participants were non-randomized when assigned to the control or intervention group. We chose not to use medicine as criteria of participant recruitment. We chose not to consider taking medicine for fat or carbohydrate metabolism as exclusion criteria. We excluded data of participants who left in the middle of the study. Data of 35 males were finally analyzed.

We chose not to consider taking medicine for fat or carbohydrate metabolism as exclusion criteria. We excluded data of participants (*n* = 23) who left in the middle of the study. Data of 35 males (mean age, 47.2 ± 7.9 years) were finally included in our analysis (Figure 1).

### Ethics

The University of Shizuoka Review Board approved the study protocol, and all selected workers provided written informed consent for their participation (No. 20–5).

### Study design

This intervention study was a non-randomized controlled trial. Participants were able to self-select the control or intervention group. The intervention group received a Japanese-style healthy lunch at their workplace cafeteria for 3 months. The total intake number of a Japanese-style healthy lunch menu is 61 times. The control group consumed their habitual lunches without restriction and only nutrient contents were assessed. The weekend dietary habits of all the participants were also unrestricted.

### Concept of a Japanese-style healthy lunch menu

The energy and nutrition contents of the provided Japanese-style healthy lunch menu were based on the Guidelines for the Diagnosis and Prevention of Atherosclerotic Cardiovascular Diseases (Japan Atherosclerosis Society, 2007) [15]. The criteria for vegetable intake matched those recommended by Healthy Japan 21. We calculated the nutritive value of the healthy lunch using the standard body type described in the results of the 2006 National Health and Nutrition survey in Japan [7]. We designed the Japanese-style healthy lunch menu to provide balanced nutrition and sufficient vegetable consumption over the course of three months (600 kcal ≤ Energy < 650 kcal, Fat < 18 g, Cholesterol ≤ 100 mg, Fiber ≥ 8 g, Total vegetables ≥ 130 g, Sodium chloride equivalent ≤ 3.8 g). The cook of the work cafeteria cooked the menu which we made a nutrient calculation.

### Nutrition assessment

We assessed the participants before and after the study intervention (consumption of Japanese healthy-style lunches for 3 months) in terms of the following items: anthropometric data, blood parameters, and dietary intake. All participants fasted after 8:00 p.m. on the day before the anthropometric measurements and blood collection. Weight and BFP (%) were measured using a BODY FAT ANALYZER TBF-215 (Tanita Co. Ltd., Tokyo, Japan). Height and weight were measured while standing barefoot and wearing light clothing. Abdominal circumference was measured after exhalation at the position of the navel by the same staff member. Blood pressure was measured using a Medinote HEM-5001 digital automatic sphygmomanometer (Omron Health Care Co. Ltd., Kyoto, Japan).

### Blood analysis

A nurse collected blood from fasting participants early in the morning. Blood was placed in vacuum tubes containing EDTA-2Na and trasylol for the active ghrelin and desacyl ghrelin analyses and into vacuum tubes containing heparin otherwise. Plasma was promptly collected by centrifugation at 1200 × *g* for 15 min at 4°C for active ghrelin and desacyl ghrelin analyses, placed in microtubes, mixed with hydrochloric acid (10% v/v), and stored at -80°C. Plasma active ghrelin and desacyl ghrelin levels were measured using ELISA kits (Mitsubishi Kagaku Latron Inc., Tokyo, Japan). We measured serum levels for T-Chol, LDL-Chol, HDL-Chol, TG, glucose, HbA_1C_ and leptin.

Serum was separated by centrifugation at 1200 × *g* and 4°C for 15 min after blood collection. Serum T-Chol, LDL-Chol, HDL-Chol, TG, glucose and HbA_1C_ levels were measured at SRL Inc. (Tokyo, Shizuoka, Japan). Serum leptin levels were measured using Human Leptin Quantikine ELISA kits (R&D Systems, Inc., Minneapolis, MN, USA).

### Dietary intake

Dietary intake was assessed using a 24-h dietary recall at baseline and after 3 months. We collected data regarding daily total intake for the day before assessment. Participants were instructed to partake in a weekday normal diet without events such as the ceremonial occasion beforehand on the day of the diet survey. The diet was documented by RD for precision and consistency. The participants answered questions about typical daily food intake, frequency of eating during the day, and variety and brand of consumed foods. Nutrition and food intake was analyzed using EXCEL Eiyo-kun software version 5.0 (Kenpaku-sha Co. Ltd., Tokyo, Japan).

### Statistical analysis

Data were analyzed using SPSS software (version 18.0, SPSS Inc., Chicago, IL, USA). Normalcy of the data was assessed using the Shapiro–Wilk test. Statistical differences were assessed using either comparison between the control and intervention groups at baseline parameters analyzed by independent sample t-test (case of parametric data) or Mann–Whitney test (case of non-parametric data). Statistical differences between the baseline parameters and the parameters after 3 months were analyzed by paired t-test (parametric data) or Wilcoxon’s signed rank test (non-parametric data). The intervention group was divided into two groups for analysis (Japanese-style healthy lunch menu intake frequency of < 50%ile and Japanese-style healthy lunch menu intake frequency of ≥ 50%ile). The group with an intake frequency of <50%ile ate the Japanese-style healthy lunch menu less than 50 times out of the total 61 times. The group with an intake frequency of ≥ 50%ile ate the Japanese-style healthy lunch menu more than 51 times out of the total 61 times.

The significance level was set at *p* < 0.05. All data are described as means ± standard deviation.

## Results

### Baseline characteristics of participants

Table 1 shows anthropometric, blood marker, and dietary intake data. Total- Cholesterol (T-Chol) levels were significantly higher in the intervention group than in the control group. On the other hand, significant differences in other blood markers or food intake were not observed between the two groups.

**Table 1:** Baseline characteristics of participants

**Characteristics**	**Control group**	**Intervention group**	**p value**	**All**
	**(n = 7)**	**(n = 28)**		**(n = 35)**
Age (y)*	42.1 ± 4.5	48.5 ± 8.1	0.013	47.2 ± 7.9
Height (cm)*	174.3 ± 4.4	168.0 ± 5.8	0.012	169.3 ± 6.0
Weight (kg)†	70.4 ± 13.1	71.8 ± 11.3	0.773	71.5 ± 11.5
BMI (kg/m^2^)*	23.1 ± 3.9	25.4 ± 3.3	0.130	24.9 ± 3.5
BFP (%)†	23.6 ± 6.5	26.1 ± 5.8	0.208	25.6 ± 5.9
SBP (mmHg)†	127.3 ± 10.0	138.8 ± 14.1	0.117	136.5 ± 14.0
DBP (mmHg)*	81.3 ± 7.4	89.3 ± 11.0	0.080	87.7 ± 10.8
Waist Circumference (cm)*	85.2 ± 11.6	88.3 ± 9.2	0.449	87.7 ± 9.7
T-Chol (mg/dL)*	185 ± 32	215 ± 27	0.014	209 ± 30
LDL-Chol (mg/dL)†	105 ± 44	131 ± 31	0.194	126 ± 35
HDL-Chol (mg/dL)*	49 ± 17	56 ± 10	0.157	55 ± 12
Triacylglycerol (mg/dL)†	240 ± 374	158 ± 95	0.343	175 ± 182
Glucose (mg/dL)†	114 ± 18	114 ± 24	0.805	114 ± 23
Hemoglobin A1C (%)†	5.0 ± 0.3	5.4 ± 0.7	0.120	5.3 ± 0.6
Active ghrelin (fmol/mL)†	3.5 ± 5.7	1.7 ± 4.6	0.614	2.0 ± 4.8
Desacyl ghrelin (fmol/mL)†	133.2 ± 161.2	62.0 ± 107.2	0.331	76.3 ± 120.6
Leptin (pg/mL)†	1692 ± 1903	2574 ± 2134	0.201	2398 ± 2093
Energy (kcal)*	2206 ± 473	2188 ± 556	0.937	2191 ± 534
Protein (g)*	75.6 ± 14.9	76.8 ± 23.0	0.892	76.6 ± 21.4
Fat (g)*	64.7 ± 15.0	67.1 ± 23.5	0.792	66.6 ± 21.9
Carbohydrate (g)*	310.2 ± 122.8	308.6 ± 93.1	0.969	308.9 ± 97.7
Salt (g)*	11.3 ± 3.3	12.0 ± 3.8	0.648	11.9 ± 3.7
Soluble dietary fiber (g)†	3.0 ± 0.7	3.4 ± 1.6	0.680	3.4 ± 1.5
Insoluble dietary fiber (g)*	12.0 ± 3.0	11.1 ± 4.0	0.599	11.3 ± 3.8
Total dietary fiber (g)*	16.7 ± 6.4	15.9 ± 7.0	0.256	16.0 ± 6.8
Green and yellow vegetable (g)†	115.0 ± 102.0	92.0 ± 74.0	0.726	96.6 ± 79.2
Other vegetable (g)*	240.1 ± 128.5	182.1 ± 119.8	0.267	193.7 ± 121.9
Total vegetable (g)*	355.1 ± 156.1	274.1 ± 168.1	0.256	290.3 ± 166.8

Data shows mean ± S.D.

*Abbreviations*: *BMI* Body mass index, *BFP* Body fat percentage, *SBP* Systolic blood pressure, *DBP* diastolic blood pressure, *T-Chol* Total cholesterol, *LDL-Chol* Low-density lipoprotein cholesterol, *HDL-Chol* high-density lipoprotein cholesterol.

*p stands for the difference between the control and intervention groups at baseline parameters analyzed by the independent sample t test. †*p* stands for the difference between the control and intervention groups at baseline parameters analyzed by the Mann–Whitney test.

Difference was considered significant at *p* < 0.05.

The frequency of metabolic syndrome and an abdominal circumference of ≥ 85 cm were 36.8% and 55.3%, respectively. Participants having an abdominal circumference of ≥85 cm weighed more, had a higher body mass index (BMI), and lower serum HDL-Chol levels than the 40- to 49-year-old men described in the National Health and Nutrition survey of 2009 [7]. They also had higher serum hemoglobin A_1C_ (HbA_1C_), glucose, T-Chol, and TG levels as well as higher fat intake. Overall, at baseline, the study participants had extremely low intake of vegetables.

### Change in anthropometric data

Table 2 shows changes in anthropometric data for each group. The control group did not show significant differences in all anthropometric data between baseline and after 3 months compared with the intervention group. However, the control group displayed a tendency of increased systolic blood pressure (SBP) (127.3 versus 132.4 mmHg, *p =* 0.063).

**Table 2:** Changes in anthropometric data

			**Intervention group**				
**Characteristics**		**Control group**	***p *** **value**	**Intake frequency of <50% ile (*n*** **= 12)**	***p *** **value**	**Intake frequency of ≥50% ile (*n*** **= 16)**	***p *** **value**
	Baseline	70.4 ± 13.1		77.2 ± 13.9		67.8 ± 7.0	
Weight (kg)			0.132*		0.546*		0.330†
	After 3 months	71.5 ± 12.8		77.5 ± 13.5		67.4 ± 6.9	
	Baseline	23.1 ± 3.9		27.2 ± 3.4		24.0 ± 2.4	
BMI (kg/m^2^)			0.175*		0.618*		0.210*
	After 3 months	23.4 ± 3.8		27.3 ± 3.2		23.8 ± 2.5	
	Baseline	23.6 ± 6.5		29.1 ± 6.9		23.8 ± 3.5	
BFP (%)			0.126*		0.783†		0.019*
	After 3 months	24.1 ± 6.8		29.0 ± 7.2		22.7 ± 3.6	
	Baseline	127.3 ± 10.0		140.5 ± 13.1		137.5 ± 15.0	
SBP (mmHg)			0.063*		0.277*		0.023†
	After 3 months	132.4 ± 10.8		138.1 ± 14.9		131.9 ± 16.9	
	Baseline	81.3 ± 7.4		90.5 ± 11.9		88.4 ± 10.6	
DBP (mmHg)			0.506*		0.000*		0.001*
	After 3 months	80.3 ± 7.4		86.3 ± 11.4		80.8 ± 8.7	
	Baseline	85.2 ± 11.6		91.8 ± 10.8		85.7 ± 7.1	
WC (cm)			0.861*		0.307*		0.129*
	After 3 months	85.0 ± 13.2		91.0 ± 10.9		84.8 ± 7.4	

Data shows mean ± S.D.

*Abbreviations*: *BMI* Body mass index, *BFP* Body fat percentage, *SBP* Systolic blood pressure, *DBP* Diastolic blood pressure, *WC* Waist circumference.

*p stands for the difference between the baseline parameters and the parameters after 3 months analyzed by the paired t-test.

†*p* stands for the difference between the baseline parameters and the parameters after 3 months analyzed by Wilcoxon’s signed rank test.

Difference was considered significant at *p* < 0.05.

For our analysis, we instituted an eating rate cutoff value at the 50%ile. Diastolic blood pressure (DBP) significantly decreased after 3 months compared with the baseline in the Japanese-style healthy lunch menu intake frequency < 50%ile group, (90.5 ± 11.9 versus 86.3 ± 11.4, *p* = 0.000). In addition, body fat percentage (BFP), SBP, and DBP significantly decreased after 3 months compared with the baseline in the Japanese-style healthy lunch menu intake frequency ≥ 50%ile group (BFP: 23.8 ± 3.5 versus 22.7 ± 3.6, *p* = 0.019; SBP: 137.5 ± 15.0 versus 131.9 ± 16.9, *p* = 0.023; DBP: 88.4 ± 10.6 versus 80.8 ± 8.7, *p* = 0.001). BFP and blood pressure decreased in the participants consuming the Japanese-style healthy lunch at a high frequency.

### Blood parameters

Table 3 shows changes in blood parameters for each group. HbA_1C_ levels in the control group had significantly increased after 3 months compared with the baseline (4.99% ± 0.29% versus 5.13% ± 0.21%, *p* < 0.05). In contrast, serum T-Chol, low-density lipoprotein (LDL-Chol), levels in the Japanese-style healthy lunch menu intake frequency ≥ 50%ile group significantly decreased (T-Chol: 211 ± 27 mg/dL versus 199 ± 22 mg/dL, *p* = 0.006; LDL-Chol: 127 ± 31 mg/dL versus 116 ± 25 mg/dL, *p =* 0.010). Furthermore, plasma active ghrelin and desacyl ghrelin levels significantly increased after 3 months compared with the baseline (active ghrelin: 1.9 ± 5.9 fmol/mL versus 5.3 ± 8.4 fmol/mL, *p =* 0.001; desacyl ghrelin: 77.4 ± 135.4 fmol/mL versus 115.7 ± 180.7 fmol/mL). In the Japanese-style healthy lunch menu intake frequency < 50%ile group, plasma active ghrelin and desacyl ghrelin levels significantly increased after 3 months compared with the baseline (active ghrelin: 1.4 ± 2.0 fmol/mL versus 3.8 ± 3.9 fmol/mL, *p =* 0.008; desacyl ghrelin: 41.6 ± 49.0 fmol/mL versus 101.4 ± 89.3 fmol/mL). Leptin levels after 3 months significantly decreased compared with the baseline in both intervention groups. Serum lipid levels decreased and eating-related hormone levels improved in the participants consuming the Japanese-style healthy lunch at a high frequency.

**Table 3:** Change in blood parameters

			**Intervention group**				
**Characteristics**		**Control group (*n*** **= 7)**	***p *** **value**	**Intake frequency of <50% ile (*n*** **= 12)**	***p *** **value**	**Intake frequency of ≥50% ile (*n*** **= 16)**	***p *** **value**
	Baseline	185 ± 32		222 ± 26		211 ± 27	
T-Chol (mg/dL)			0.191*		0.229*		0.006*
	After 3 months	199 ± 40		215 ± 28		199 ± 22	
	Baseline	105 ± 44		136 ± 30		127 ± 31	
LDL-Chol (mg/dL)			0.292*		0.437*		0.010*
	After 3 months	109 ± 45		132 ± 26		116 ± 25	
	Baseline	49 ± 17		53 ± 7		59 ± 11	
HDL-Chol (mg/dL)			0.522*		0.287*		0.073*
	After 3 months	49 ± 16		51 ± 8		56 ± 15	
	Baseline	240 ± 374		208 ± 116		121 ± 55	
Triacylglycerol (mg/dL)			0.173†		0.136†		0.408†
	After 3 months	289 ± 436		181 ± 67		115 ± 51	
	Baseline	113 ± 18		108 ± 19		118 ± 28	
Glucose (mg/dL)			0.735†		0.583†		0.255†
	After 3 months	112 ± 21		107 ± 16		111 ± 18	
	Baseline	5.0 ± 0.3		5.3 ± 0.5		5.5 ± 0.8	
Hemoglobin A1C (%)			0.041†		0.033†		0.529†
	After 3 months	5.1 ± 0.2		5.4 ± 0.5		5.5 ± 0.7	
	Baseline	3.5 ± 5.7		1.4 ± 2.0		1.9 ± 5.9	
Active ghrelin (fmol/mL)			0.128†		0.008†		.001†
	After 3 months	6.4 ± 7.4		3.8 ± 3.9		5.3 ± 8.4	
	Baseline	133.2 ± 161.2		41.6 ± 49.0		77.4 ± 135.4	
Desacyl ghrelin (fmol/mL)			1.000†		0.002†		0.001†
	After 3 months	108.0 ± 67.7		101.4 ± 89.3		115.7 ± 180.7	
	Baseline	1692 ± 1903		3611 ± 2786		1797 ± 999	
Leptin (pg/mL)			0.074*		0.001*		0.000†
	After 3 months	308 ± 219		610 ± 391		313 ± 158	

Data shows mean ± S.D. *Abbreviations*: *T-Chol* Total cholesterol, *LDL-Chol* Low-density lipoprotein cholesterol, *HDL-Chol* high-density lipoprotein cholesterol. *p stands for the difference between the baseline parameters and the parameters after 3 months analyzed by the paired t-test. †*p* stands for the difference between the baseline parameters and the parameters after 3 months analyzed by Wilcoxon’s signed rank test. Difference was considered significant at *p* < 0.05.

### Changes in nutrition intake determined by 24-h dietary recall

Table 4 show changes in nutrition intake for each group determined by 24-h dietary recall. The "other vegetable" intake in the control group showed a significant decrease after 3 months compared with the baseline (240.1 g ± 128.5 g versus 96.4 g ± 64.7 g, *p* = 0.015). On the other hand, energy and carbohydrate intake significantly decreased after 3 months compared with the baseline in the Japanese-style healthy lunch menu intake frequency < 50%ile group (energy: 2554 ± 392 kcal versus 2104 ± 393 kcal, *p =* 0.042; carbohydrate: 359.6 ± 85.2 g versus 295.8 ± 45.3 g). Furthermore, total dietary fiber and total vegetables in the Japanese-style healthy lunch menu intake frequency ≥ 50%ile group significantly increased (total dietary fiber: 15.3 ± 5.2 g versus 30.4 ± 20.9 g, *p* = 0.047; total vegetables: 292.4 ± 146.6 g versus 411.1 ± 155.9 g, *p =* 0.035). Vegetable intake increased in the intervention group with the use of the Japanese-style healthy lunch menu.

**Table 4:** Changes in dietary intake

				**Intervention group**			
**Characteristics**		**Control group (*n*** **= 7)**	***p *** **value**	**Intake frequency of <50% ile (*n*** **= 12)**	***p *** **value**	**Intake frequency of ≥50% ile (*n*** **= 16)**	***p *** **value**
	Baseline	2206 ± 473		2554 ± 392		1938 ± 517	
Energy (kcal)			0.336		0.042		0.543
	After 3 months	2423 ± 548		2104 ± 393		2040 ± 403	
	Baseline	75.6 ± 14.9		86.4 ± 23.1		68.2 ± 21.0	
Protein (g)			0.266		0.765		0.028
	After 3 months	84.4 ± 13.6		82.9 ± 25.6		90.9 ± 30.0	
	Baseline	64.7 ± 15.0		78.8 ± 19.4		55.7 ± 15.5	
Fat (g)			0.774		0.151		0.286
	After 3 months	68.3 ± 31.2		59.5 ± 24.7		49.1 ± 13.1	
	Baseline	310.2 ± 122.8		359.6 ± 85.2		284.8 ± 91.3	
Carbohydrate (g)			0.286		0.049		0.386
	After 3 months	345.2 ± 116.2		295.8 ± 45.3		308.1 ± 66.1	
	Baseline	11.3 ± 3.3		12.8 ± 4.1		11.2 ± 4.0	
Salt (g)			0.823		0.723		0.426
	After 3 months	11.6 ± 2.3		12.2 ± 5.0		12.5 ± 5.3	
	Baseline	16.7 ± 6.4		18.2 ± 9.0		15.3 ± 5.2	
Total dietary fiber (g)			0.912		0.661		0.047
	After 3 months	17.1 ± 7.7		20.8 ± 14.9		30.4 ± 20.9	
	Baseline	355.1 ± 156.1		298.4 ± 203.5		292.4 ± 146.6	
Total vegetables (g)			0.210		0.232		0.035
	After 3 months	273.8 ± 152.2		211.0 ± 110.0		411.1 ± 155.9	

Data shows mean ± S.D.

*p* stands for the difference between the baseline parameters and the parameters after 3 months analyzed by the paired t-test.

Difference was considered significant at *p* < 0.05.

## Discussion

Specific medical examinations and health guidance to prevent and/or improve metabolic syndrome have been implemented by insurers in Japan since 2008 to stem the increase in lifestyle-related diseases. However, the objectives of reducing health care costs, reducing abdominal obesity, and preventing metabolic syndrome have not yet been realized. The Healthy Japan 21 advocated by the Japanese Ministry of Health, Labour and Welfare (2000) recommends that adults ingest ≥ 350 g of vegetables per day [6]. Therefore, changes in food consumption patterns and environment must be incorporated into primary countermeasures against lifestyle-related diseases. Thus, we provided a Japanese-style healthy lunch menu daily for 3 months at a workplace cafeteria to examine the ability of consumption of this style of meal to prevent and/or improve metabolic syndrome.

The intervention group displayed reduced blood pressure and serum lipid markers after 3 months compared with the baseline. These improvements were observed particularly in cases where the intake frequency of the Japanese-style healthy lunch was high. Furthermore, plasma active ghrelin and desacyl ghrelin levels, which are related to appetite, increased compared with the baseline. These results mainly seem attributable to increased vegetable intake. In contrast with the intervention group, the control group showed unchanged biochemical markers and anthropometric data throughout the time frame of the study. Hung et al. reported that high fruit and vegetable intake helps to modestly reduce the risk of major chronic disease, and their findings support the recommendation of consuming at least five daily servings of fruits and vegetables [16]. Increasing fruit and vegetable intake appears to confer a benefit primarily of a lower risk of CVD but not of cancer. The effect of the present intervention on cancer prevention is uncertain.

However, an increase in dietary fiber and vitamins could partly explain the lower blood pressure and decreased serum lipid markers observed in the intervention group. Fiber is a dietary factor that has received substantial attention [17,18]. Burton-Freeman reported that dietary fiber functions as an energy intake regulator [19]. Furthermore, the contents of the provided healthy lunches support the claim that the intervention group had an increase in total and soluble dietary fiber intake after compared with before the intervention. Liu et al. suggested that higher intake of fruits and vegetables can protect against CVD, and their findings support current dietary guidelines that promote increased fruit and vegetable intake [20]. They also reported that higher intake of dark yellow and green leafy vegetables can help to prevent type 2 diabetes among overweight women [20].

In addition, we surmised that a high-fiber diet increases the amount of mastication. Yamazaki et al. reported that higher masticatory performance and eating slowly helps prevent diabetes [21]. Their results imply that higher mastication can decrease blood glucose and HbA_1C_ levels. However, the present study did not find any changes in related blood parameters. We believe that blood parameters can be improved by lengthening the period of intervention and increasing the frequency with which individuals choose the foods offered on healthy menus.

In the intervention group, plasma active ghrelin and desacyl ghrelin levels significantly increased after 3 months compared with the baseline.

Ghrelin is an orexigenic hormone predominantly secreted by the stomach and stimulates appetite and food intake after i.v. administration [22]. The postprandial ghrelin response depends on the nutrient composition of ingested meals, and several studies support the notion that elevated postprandial and later interdigestive ghrelin levels could contribute to recurrent hunger pangs and appetite [23]. Ghrelin is a somatotropic and orexigenic hormone that functions as an important energy metabolism regulator [22] and a physiological regulator of insulinemia and glycemia [23]. Some studies have shown that circulating plasma ghrelin levels are lower in obese children and adults than in age-matched lean control individuals [24,25]. Plasma ghrelin levels increase in individuals with a negative energy balance such as those on a low-energy diet or anti-obesity medication, or in those who exercise regularly [26,27]. Furthermore, plasma ghrelin might play a role in energy balance [27].

Horigome notably reported significant associations among hemostatic parameters, adipokines, and metabolic syndrome components among Japanese preschool children [28]. Children with BMI of ≥90%ile had significantly higher SBP and heart rates and higher blood levels of insulin and leptin [28]. They also showed higher insulin resistance by the homeostasis model assessment and lower desacyl ghrelin levels than children with BMI of <90% ile [28].

Pöykkö and Fagerberg described that ghrelin concentrations were negatively associated with fasting insulin and systolic and diastolic blood pressure in the multivariate models [29,30]. SBP/DBP in the present study was significantly reduced after 3 months compared with the baseline.

Furthermore, serum leptin levels after 3 months were significantly reduced compared with the baseline in the intervention group. Particularly in the Japanese-style healthy lunch menu intake frequency ≥ 50%ile group, BFP after 3 months significantly decreased compared with the baseline. Leptin, which is produced by adipose tissue, reduces food intake while increasing energy expenditure and positively correlates with fat mass [31,32]. Elevated leptin levels are independently correlated with increased CVD risk [33,34]. Reduced leptin levels while fasting during weight loss can be attributed to decreased leptin resistance [35]. The relatively short duration of the intervention could explain the finding that BFP and serum leptin levels decreased in the present study. In the control group, no statistically significant differences in active ghrelin, desacyl ghrelin, and leptin concentrations between baseline and after 3 months were observed. It is unknown clinically. We believe that inspection by long-term intervention in a randomized clinical trial is necessary in future to determine the effect of ghrelin and leptin concentrations.

We believe that these findings will contribute to epoch-making metabolic syndrome prevention and improvement. However, this study is limited by its structure as a small non-randomized controlled trial. Hence, we plan to implement an additional intervention study with an increased number of participants.

## Conclusions

This non-randomized controlled trial of a Japanese-style healthy lunch consumed at a workplace cafeteria aimed to prevent and/or improve metabolic syndrome among middle-aged men. The result revealed that continuing intake of a Japanese-style healthy lunch decreased blood pressure and serum lipids and increased plasma ghrelin levels. Our study results demonstrate that an intervention consisting of short-term consumption of a Japanese-style healthy lunch at a workplace cafeteria contributes to lipid metabolism regulation. These findings might lead to the design of a novel approach for improving the quality of food provision in out-of-home environments in Japan.

## Abbreviations

UNESCO: United Nations Educational, Scientific and Cultural Organization; CVD: Cardiovascular disease; TG: Triacylglycerol; HDL-Chol: High-density lipoprotein; Healthy Japan 21: The National Health Promotion Campaigns for the 21st Century; T-Chol: Total cholesterol; BMI: Body mass index; HbA_1C_: Hemoglobin A_1C_; SBP: Systolic blood pressure; DBP: Diastolic blood pressure; BFP: Body fat percentage; LDL-Chol: Low-density lipoprotein; EDTA: Ethylenediaminetetraacetate; ELISA: enzyme-linked immunosorbent assay.

## Competing interests

The authors of the manuscript declare no conflicts of interest.

## Authors’ contributions

HI, RS and TK managed the study. HI, RS and IA conducted blood analysis and dietary assessment. HI, RS and TK undertook statistical analysis. HI and TK drafted the manuscript. All authors read and approved the final manuscript.

## Authors’ information

Laboratory of Nutrition Education, Department of Food and Nutritional Sciences and Environmental Health Sciences, Graduate School of Integrated Pharmaceutical and Nutritional Sciences, University of Shizuoka, Shizuoka 422–8526, Japan.

Hiroko Inoue is a registered dietitian, Ph.D., Ryosuke Sasaki is a registered dietitian, Izumi Aiso is a pharmaceutical chemist and Toshiko Kuwano is a registered dietitian, Ph.D., Department of Food and Nutritional Sciences and Environmental Health Sciences, Graduate School of Integrated Pharmaceutical and Nutritional Sciences, University of Shizuoka.
